# 

**Published:** 1899-06

**Authors:** 


					CONTENTS.
On the Temperature in Cases of
Apoplexy, and on the Occurrence
(1) of (Edema and (2) of Loss of the
Knee-jerk in the Paralysed Limbs
in Hemiplegia.
By J. Michell
Clarke, M.A., M.D. Cantab.,
F.R.C.P
97
Fracture of the Patella.
By A. W.
Prichard, M.R.C.S.
104
Idiopathic Thrombosis of the Portal
and Contributory Veins.
By
Bertram M. H. Rogers, B.A.,
M.D., B.Ch. Oxon
107
A Case of Extreme Bradycardia.
By
R. George Fendick, M.R.C.S., and
George Parker, M.D. Cantab.
With Notes on the Eye-Symp-
toms
by r. Richardson Cross,
M.B. Lond., F.R.C.S.
113
A Case of Dislocation of the Hand
and Carpus Backwards.
By
Trafford Mitchell, M.D. Glasg.
121
Progress of the Medical Sciences:
Medicine.
R. Shingleton Smith,
M.D.
121
Surgery.
C. A. Morton
126
Obstetrics and Gynaecology.
Walter C. owayne,
131
Reviews of Books ..
138
Notes on Preparations for the Sick
160
Bristol Medlco-Chlrargical Society
161
Devon and Exeter Medical Society...
173
The Library of the Bristol Medico-
Chirurgical Society 177
Local Medical Notes
181
A Memorial to Dr. Joseph O'Dwyer...
189
The "Index Medlcus"
189
Scraps
190

				

